# Review on Human Action Recognition in Smart Living: Sensing Technology, Multimodality, Real-Time Processing, Interoperability, and Resource-Constrained Processing

**DOI:** 10.3390/s23115281

**Published:** 2023-06-02

**Authors:** Giovanni Diraco, Gabriele Rescio, Pietro Siciliano, Alessandro Leone

**Affiliations:** National Research Council of Italy, Institute for Microelectronics and Microsystems, 73100 Lecce, Italy; pietroaleardo.siciliano@cnr.it (P.S.); alessandro.leone@cnr.it (A.L.)

**Keywords:** review, human action recognition, smart living, multimodality, real-time processing, interoperability, resource-constrained processing, sensing technology, machine learning, deep learning, signal processing, smart home, smart environment, smart city, smart community, ambient assisted living

## Abstract

Smart living, a concept that has gained increasing attention in recent years, revolves around integrating advanced technologies in homes and cities to enhance the quality of life for citizens. Sensing and human action recognition are crucial aspects of this concept. Smart living applications span various domains, such as energy consumption, healthcare, transportation, and education, which greatly benefit from effective human action recognition. This field, originating from computer vision, seeks to recognize human actions and activities using not only visual data but also many other sensor modalities. This paper comprehensively reviews the literature on human action recognition in smart living environments, synthesizing the main contributions, challenges, and future research directions. This review selects five key domains, i.e., Sensing Technology, Multimodality, Real-time Processing, Interoperability, and Resource-Constrained Processing, as they encompass the critical aspects required for successfully deploying human action recognition in smart living. These domains highlight the essential role that sensing and human action recognition play in successfully developing and implementing smart living solutions. This paper serves as a valuable resource for researchers and practitioners seeking to further explore and advance the field of human action recognition in smart living.

## 1. Introduction

The smart living concept embodies a technology-driven lifestyle to elevate life quality, increase efficiency, and reduce waste. Academics and researchers have thoroughly investigated this idea, encompassing diverse dimensions, such as technology, security, health, and education [1], among others. It employs state-of-the-art Information and Communication Technology (ICT), advanced sensing technology, pervasive computing, big data analytics, and intelligent decision making to optimize energy consumption, enhance healthcare, and elevate living standards [2,3]. Closely linked to smart cities, smart living encourages citizen traits such as awareness, independence, and participation [1]. It aims to transform life and work through ICT, fostering sustainable economic growth and exceptional quality of life while conserving natural resources via collaborative governance [4]. The central idea is to generate benefits for citizens, considering their well-being and engagement [5].

Smart living technologies empower users to access and analyze personal information, such as health and living conditions [3]. Giffinger et al. [6] proposed a smart city framework with six core components: smart economy, smart people, smart governance, smart mobility, smart environment, and smart living. Integrating stakeholders, such as individuals, machines, devices, and the environment, is crucial for smart living, covering aspects such as lighting, water, traffic, parking, objects, buildings, industry, and context-based services [7]. While connectivity and information drive smart living, emphasizing enhanced living quality under sustainable conditions is essential over technological innovation [8]. As smart living evolves, adapting designs, devices, technology, and sensors becomes critical for a sustainable lifestyle [7,9].

In such a scenario, Human Action Recognition (HAR) plays a significant role in smart living, contributing to various applications such as home automation, healthcare, safety, and security. Accurately identifying and interpreting human actions allows smart living systems to offer real-time responses, delivering personalized support and assistance. In particular, this work emphasizes the domains of Sensing Technology, Multimodality, Real-time Processing, Interoperability, and Resource-Constrained Processing. These elements encapsulate the critical aspects necessary for successfully deploying HAR in smart living environments. Recognizing human actions is essential for effectively implementing smart living solutions, making it a key area of research and development to pursue an enhanced quality of life and more efficient, sustainable living spaces.

### 1.1. General Background on HAR

HAR refers to the process of recognizing and understanding human actions, which is essential for various real-world applications, such as assisted living [10], visual surveillance [11], autonomous navigation [12], video retrieval [13], human–robot interaction [14,15], and entertainment [16]. The concept of HAR encompasses various aspects, including action recognition, intention understanding, and narrative understanding [17,18]. Indeed, essentially, HAR involves identifying and interpreting human actions and environmental interactions, particularly whole-body and limb movements. Understanding these actions is crucial for predicting their effects or outcomes and inferring the performer’s intention, goal, and mental status [19].

Primary methodologies for HAR include traditional approaches, such as template matching [20], space-time features [21], and action grammars [22], as well as more recent Deep Learning (DL) techniques, such as Convolutional Neural Networks (CNNs), Recurrent Neural Networks (RNNs), and Long Short-Term Memory (LSTM) networks [23], to name a few. These methods leverage various sensing modalities, such as RGB (Red-Green-Blue) videos, skeleton data, depth data, infrared, thermal, point clouds, audio, binary readings, acceleration, gyroscope, magnetometer, radar, and Wi-Fi. Furthermore, these diverse sensing approaches provide diverse and complementary information about human actions. Visual modalities (e.g., RGB videos, skeleton data, depth data, infrared sequences, point clouds), for example, are more intuitive for representing human actions, since they are more similar to the functioning of the human visual system. In contrast, non-visual modalities (e.g., acceleration, gyroscope, magnetometer, radar, and Wi-Fi) can be used for privacy-sensitive scenarios or when visual data are insufficient or unavailable.

Data fusion techniques improve recognition models by integrating information from multiple modalities, enhancing understanding of human actions, and addressing individual modalities’ limitations. These techniques, along with action prediction, narrative understanding, and transfer learning, significantly develop HAR research, expanding its real-world application [24]. Data fusion increases the accuracy of HAR models and can be used at feature, decision, or model levels [25].

Further aspects relevant to HAR include the investigation of action prediction, where the goal is to anticipate future actions based on observed actions, and narrative understanding, which focuses on identifying the agent’s identity or social role in the context of an action [26,27]. Additionally, transfer learning and co-learning across different modalities can improve the robustness and generalizability of HAR models, enabling them to adapt to new scenarios and handle diverse data sources effectively [28,29,30].

### 1.2. HAR in Smart Living Focusing on Sensing Technology, Multimodality, Real-Time Processing, Interoperability, and Resource-Constrained Processing

HAR aims to provide smart homes and buildings with an understanding of the behaviors and routines of the occupants, allowing for improved automation and personalized services. For example, in a smart home, HAR can be used to recognize and respond to the actions of the occupants, such as opening and closing doors, turning on and off lights, and adjusting the temperature.

HAR monitors patient activities in smart healthcare and manages citizen actions in smart cities for traffic, public safety, and the environment. Effective implementation in smart living necessitates considering domains, such as Sensing Technologies, Multimodality, Real-time Processing, Interoperability, and Resource-Constrained Processing. Addressing these factors, HAR develops intelligent systems that adapt to smart living environments, enhancing individual and community life quality. The importance and impact of these HAR aspects will be elaborated on in the following sections.

**Figure 1:**
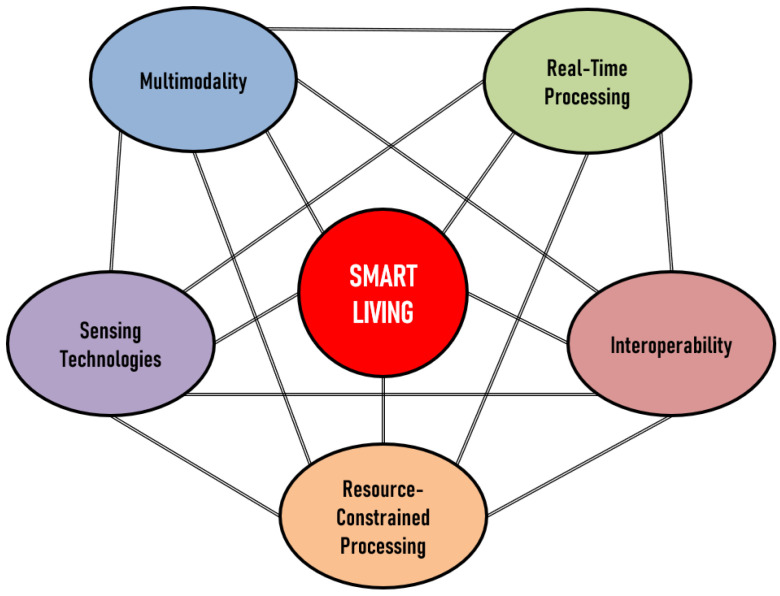
A visual representation of the interconnected domains—Sensing Technologies, Multimodality, Real-time Processing, Interoperability, and Resource-Constrained Processing—that form the foundation of a Smart Living ecosystem, highlighting the synergistic relationships among them.

In Figure 1, we can observe the representation of the five key domains, interconnected with Smart Living and sharing connections among themselves. The interconnectivity among these domains arises due to the complementary and synergistic nature of their roles in enabling and enhancing Smart Living. Each domain contributes unique capabilities and characteristics that, when combined, result in more efficient, user-centric, and intelligent systems.

This review paper will comprehensively analyze the current state-of-the-art HAR within smart living. The remainder of the paper is organized as follows: First, we delve into the existing review works in the field, identifying the gaps and motivating the need for this comprehensive study. We then describe the search and inclusion criteria for selecting the relevant literature. Section 3 presents an overview of the common publicly available datasets used in the studies, followed by a discussion of the widely used performance metrics for evaluating Machine Learning (ML) algorithms in Section 4. Section 5 explores various aspects of HAR in smart living through the proposed smart living Temple framework. This framework allows us to examine the interplay between different aspects of HAR and smart living, such as sensing modalities, feature extraction techniques, and classification methods.

Section 6 presents a critical discussion addressing potential concerns and challenges related to the smart living Temple, offering valuable insights for researchers and practitioners. Finally, we conclude the paper in Section 7, summarizing our findings and providing some closing considerations for future research and development in HAR in smart living applications.

## 2. Review of Related Works and Rationale for This Comprehensive Study

HAR has gained significant attention as a crucial research area in various application contexts in recent years. This increasing importance has led to the publication of numerous review studies focused on different aspects of the field. Based on a thorough examination of existing literature, it can be concluded that existing survey and review studies predominantly fall into one of the following categories: either they provide a general overview of the field [31,32], or they focus on specific aspects, such as ML, DL, and hardware architectures [23,33,34,35], sensing [36,37,38], or computer vision [39,40,41]. To the authors’ best knowledge, it is important to note that no studies specifically centered on smart living comprehensively analyze the current literature through the aforementioned key domains essential for smart living solutions. This observation requires a comprehensive literature review on HAR for smart living. Such a review would facilitate a more nuanced understanding of the state of the art in this domain, ultimately fostering the advancement of innovative, effective, and efficient smart living technologies.

For this review, a thorough literature analysis was conducted by examining 511 documents identified using a targeted Scopus query. The query was designed to capture many relevant papers by incorporating specific keywords related to human activity recognition and smart Living. The query utilized the following structure:

*TITLE (action OR activity OR activities) AND TITLE (recognition OR classification OR classifying OR recognize OR classified OR classifier OR detector OR detecting OR discriminating OR discrimination) AND TITLE-ABS-KEY (“smart home” OR “smart building” OR “smart environment” OR “smart space” OR “smart living” OR “smart city” OR “smart cities” OR “assisted living” OR “ambient intelligence” OR “smart ambient”) AND PUBYEAR > 2019.*

The query searched for articles with titles containing terms related to actions or activities and their recognition, classification, or detection. Furthermore, the search was refined to include articles with title-abstract-keywords related to various smart living contexts, such as smart homes, smart buildings, smart environments, smart cities, and ambient intelligence, among others. Additionally, the query focused on publications from 2020 onwards to ensure that the analysis considered recent advancements in the field.

The primary inclusion criterion for selecting a paper in this review was its contribution to one or more of the key domains of smart Living mentioned earlier. This approach allowed for the compilation of a comprehensive and relevant set of literature, which forms the basis for an informed and insightful analysis of human activity recognition in the context of smart living.

## 3. Common Publicly Available Datasets

While numerous publicly available datasets are commonly used for HAR, it is crucial to recognize that smart living applications have specific requirements that these datasets may need to address fully. One of the key considerations when selecting a dataset for smart living applications is the type of human action included in the dataset. Human actions should be relevant to the specific context of smart living and reflect individuals’ daily routines and tasks within their homes, workplaces, or urban environments. This ensures that the HAR models developed from these datasets are tailored to the unique needs of smart living solutions.

Another crucial factor to consider is the number of subjects involved in the dataset. A diverse range of subjects, with varying ages, genders, and physical abilities, can provide a more comprehensive representation of human activities. This diversity helps develop more robust and generalizable HAR models that cater to the broader population and are adaptable to different individuals and situations.

Other aspects that should be considered when selecting a dataset for HAR in smart living include the quality of data, the number of sensors used, the positioning of these sensors, and the duration of the recorded activities. These factors can significantly impact the performance and reliability of HAR models, making it essential to consider them when choosing the most suitable dataset for a given application.

In this review, we have carefully selected several relevant datasets widely adopted by the research community for Human Activity Recognition (HAR) studies. These datasets include Opportunity [42], PAMAP2 [43], UniMiB [44], CASAS: Aruba [45], CASAS: Cairo [46], CASAS: Kyoto [47], CASAS: Kyoto Multiresident [48], CASAS: Milan [47], CASAS: Tokyo [49], CASAS: Tulum [47], WISDM [50], ExtraSensory [51], USC-HAD [52], Skoda [53], UP-Fall [54], UK-DALE [55], MARBLE [56], KTH [57], Weizmann [58], UCF Sports Action [59], SisFall [60], LARa [61], UCI-HAR [62], UT_complex [63], UTD-MHAD [64], and UCI-SBHAR [65].

An overview of the different datasets used by the review works is provided in Table 1.

## 4. Performance Metrics

In evaluating the performance of classification algorithms, several key metrics are commonly used: Accuracy (*A*), Recall (*R*), Precision (*P*), F1-score (*F*1*S*), macro-F1-score (*mF*1*S*), and Specificity (*SP*). Accuracy is defined as follows:

(1) $$A=\frac{TP+TN}{TP+TN+FP+FN},$$

which measures the proportion of correct predictions made by the model out of the total predictions. Recall is defined as follows:

(2) $$R=\frac{TP}{TP+FN},$$

which is also known as sensitivity or true positive rate, and quantifies the fraction of relevant instances that have been retrieved. Precision is defined as:

(3) $$P=\frac{TP}{TP+FP},$$

which represents the proportion of true positives among the predicted positives. The F1-score is defined as follows:

(4) $$F1S=2\times \frac{P\times R}{P+R},$$

which is the harmonic mean of precision and recall, balancing their trade-offs.

The macro-F1-score is defined as follows

(5) $$mF1S=\frac{1}{N}\sum_{i=1}^{N}F1S_{i},$$

which calculates the average of the F1-scores for each class, treating all classes equally regardless of their size. Finally, Specificity is defined as:

(6) $$SP=\frac{TN}{TN+FP},$$

which gauges the proportion of true negatives among the predicted negatives, reflecting the model’s ability to identify negative instances correctly.

Regarding the symbols above, *TP*, *TN*, *FP*, and *FN* commonly represent different outcomes in a binary classification problem. They are defined as follows:

- *TP*: True Positives—the number of positive cases correctly identified by a classifier;
- *TN*: True Negatives—the number of negative cases correctly identified as negative by a classifier;
- *FP*: False Positives—the number of negative cases incorrectly identified as positive by a classifier;
- *FN*: False Negatives—the number of positive cases incorrectly identified as negative by a classifier.

## 5. Recent State of the Art on HAR in Smart Living

This section provides an in-depth analysis of various aspects of HAR within smart living as a cutting-edge field revolutionizing how we interact with our environments. The critical domains of this analysis include Sensing Technologies, Multimodality, Real-time Processing, Interoperability, and Resource-Constrained Processing, which collectively contribute to creating seamless, adaptive, and responsive living spaces.

Each domain represents a crucial aspect of HAR, and together, they support the overarching goal of creating seamless, adaptive, and secure living spaces. Through these domains, the suggested framework encapsulates the vital elements contributing to the rapid advancements in HAR. In the following discussion, we will examine each domain of the framework in detail, shedding light on their significance and interconnections, ultimately providing a comprehensive review of HAR literature as an integral aspect of the future of smart living.

The process of human activity recognition in smart living can be visualized as a progression from data acquisition to knowledge extraction and utilization. This process begins with the sensing technology, which forms the basis for data collection. Multimodality is then used to enhance the data collection process by combining different sensing modalities. The collected data are then subjected to real-time processing, which is necessary for providing timely and responsive services in smart living applications. Interoperability is crucial for integrating various devices and systems, enabling seamless communication and data sharing. Finally, all these processes need to be performed under resource-constrained conditions, given the limitations in processing power, energy, and memory in many smart living devices. In the following sections, we will delve into each domain in the presented order.

### 5.1. Sensing Technologies

Sensors play a central role in developing smart living applications, enabling the recognition of human actions to improve the overall quality of life. These applications, as discussed in the previous sections, aim to provide increased comfort, safety, and energy efficiency by understanding and adapting to the needs and preferences of their users. Sensing technologies are crucial for identifying and interpreting human actions, and their advances directly impact the performance and effectiveness of HAR systems in smart living environments. According to the revised literature, sensors can be categorized based on the sensing principle or their operational position. When classifying sensors according to the sensing principle, we can identify several subcategories:

Mechanical sensors include:

- Inertial sensors (accelerometers and gyroscopes) that measure acceleration and angular velocity to detect motion and orientation.
- Pressure sensors that measure force per unit area, enabling the detection of physical interactions, such as touch or contact between objects.
- Acoustic sensors that capture sound waves to identify events such as footsteps, speech, or glass breaking.
- Vibration sensors that detect oscillations and vibrations in structures or objects can indicate various activities or events.
- Ultrasound sensors that use high-frequency sound waves to measure distance or detect movement are often employed in obstacle detection and proximity sensing.
- Contact switch sensors that detect a physical connection’s open or closed state, such as doors or windows.

Electromagnetic sensors include:

- Magnetic sensors that detect changes in magnetic fields are often used for tracking the movement or orientation of objects.
- Visual spectrum sensors, such as cameras, capture images and videos to recognize activities, gestures, and facial expressions.
- Infrared or near-infrared sensors, including cameras, Passive Infrared (PIR) sensors, and IR arrays, can detect heat signatures, enabling motion detection and human presence recognition.
- RF systems such as WiFi and radar utilize wireless signals to sense movement, location, and even breathing patterns.

An alternative approach to categorizing sensors, based on their underlying sensing principles, involves classifying them according to the types of waves they utilize:

- Visible spectrum sensors (e.g., cameras) that capture images in the range of wavelengths perceivable by the human eye.
- Infrared sensors that detect thermal radiation emitted by objects help identify human presence and motion.
- Radio-frequency sensors that employ wireless signals to track movement, proximity, and location.
- Mechanical wave/vibration sensors, including audio, sonar, and inertial sensors, which capture sound waves, underwater echoes, or physical oscillations, respectively.

Besides the previous ways to categorize sensors, an efficient approach is to classify them by operational position. By examining sensors through this lens, we consider where sensors are placed, worn on the body, or attached to objects, which is a more practical and application-oriented categorization method. When categorizing sensors by operational position, we can distinguish the following groups:

- Environmental sensors, which monitor physical parameters such as atmospheric pressure, temperature, humidity [66], and open/close states of doors or windows, pressure force sensors installed on the floor or chairs to detect people’s presence [67].
- Ambient sensors, including cameras [68], microphones [69], radio-frequency (radar, WiFi) [69], and motion detectors [70], which capture information about the surrounding environment to identify activities or events [71].
- Object-attached sensors, such as inertial sensors mounted on everyday objects [72], which track the movement or usage of these objects [42].
- Body-worn sensors, predominantly inertial sensors, that are attached to the human body to monitor activities [73], gestures [74], and postures [75], but also physiological sensors measuring neurovegetative parameters, such as heart rate, respiration rate, and blood pressure [76]. Wearable sensors can also be employed for comprehensive health monitoring, enabling continuous tracking of vital signs [77].

The type of sensor used in HAR applications significantly impacts the system’s performance and capabilities. For instance, while some sensors provide high accuracy and rich contextual information, they may also require more computational resources or power consumption, making them less suitable for specific applications. On the other hand, some sensors are more energy-efficient and lightweight (e.g., binary sensors), which may be desirable in specific scenarios, but may also come at the cost of reduced accuracy or limited contextual information.

Wearable sensors, particularly inertial sensors, are among the most commonly used in HAR applications within the smart living context. Generally, actions detected using wearable sensors tend to be coarse, such as walking, running, sitting, or standing [78]. While these sensors can accurately recognize basic actions, more complex or nuanced actions may be challenging to detect.

Most studies in this field focus on feature extraction and classification techniques to improve HAR accuracy [79]. However, more emphasis on system architecture issues often needed, such as low power consumption, lightweight algorithms, and embedded processing [80]. While real-time processing is frequently addressed, other critical aspects of HAR system design may need more attention [81].

Body position is a crucial parameter for wearable sensors, as it influences the quality and reliability of the collected data. For example, the accuracy of a sensor can be affected by its position on the body, the orientation of the sensor, and any potential interference caused by clothing or other factors [75,82]. Therefore, wearable sensors are often investigated with environmental sensors, which can provide complementary information to enhance HAR performance [81].

The acceptability of HAR sensing technologies hinges on perceived intrusiveness. Cameras raise privacy concerns, while wearables may affect comfort. RF sensing, preserving privacy and offering valuable activity data, is a promising HAR modality [83]. WiFi [84] and radar sensors [85] offer non-intrusive monitoring without cameras or wearables. While RF sensing detects coarse actions, its recognition of fine actions is limited [86]. Overcoming this necessitates enhancing RF sensing via research, signal processing advancements, ML algorithms, and sensor fusion [87]. Multimodal sensing approaches can also provide a comprehensive understanding of activities.

Besides RF sensing, researchers have explored various approaches to positioning sensors around smart environments to enhance human activity recognition and individual identification. One notable example is the Triboelectric Nanogenerator (TENG)-based gait sensor system [88]. The TENG-based gait sensor system utilizes triboelectric nanogenerators to detect mechanical motions through electrical signals, such as human steps. By embedding these sensors into a smart carpet on the floor, this method offers a non-intrusive and reliable means of monitoring human activities and recognizing individual walking patterns, overcoming traditional sensing technologies’ limitations and privacy concerns.

The works discussed in this section are summarized in Table 2.

Having discussed the various aspects of sensing technology, we now turn our attention to multimodality, which enhances the data collection process by combining different sensing modalities.

### 5.2. Multimodality

Multimodality is a critical aspect of smart living, encompassing a variety of applications, such as health monitoring, human–object interaction, and smart homes. It effectively integrates various sensing modalities, including wearable devices and environmental sensors, to achieve accurate and reliable HAR. Although significant advancements have been made in recent years using CNNs, LSTM networks, transformer networks, and various hybrid models, challenges persist in effectively modeling spatial-temporal dependencies of sensor signals and addressing the distinct contributions of different sensing modalities in complex environments [89,90].

To tackle these challenges, Xiao et al. [91] proposed a self-attention-based Two-stream Transformer Network (TTN). The TTN aims to model the temporal-spatial dependencies for multimodal HAR by introducing an attention block to evaluate the recognition performance of all sensor axis readings. The model incorporates a two-stream structure consisting of temporal and spatial channels, which extract sensor-over-time and time-over-sensor features, respectively. While the authors primarily focuses on wearable sensing, the concepts and techniques presented can potentially be extended to environmental sensing. A more comprehensive and robust human activity recognition system can be developed by considering various sensing modalities, such as cameras, microphones, and other ambient sensors.

In the paper by Bocus et al. [24], the authors present a comprehensive multimodal dataset designed for passive HAR and localization techniques using synchronized Radio Frequency (RF) devices and vision-based sensors. The dataset is unique because it incorporates multiple synchronized modalities, including Channel State Information (CSI) from a WiFi Network Interface Card (NIC), passive WiFi radar based on a Software Defined Radio (SDR) platform, Ultra-Wideband (UWB) signals, and vision/infrared data from Kinect sensors. The dataset consists of approximately 8 hours of annotated measurements collected across two rooms from six participants performing six daily activities.

Islam et al. [92] presented a DL-based fusion approach for multimodal HAR in smart healthcare applications. The fusion technique is designed to handle different types of data collected from wearable and stationary devices (i.e., environmental sensors). The authors utilize CNNs to extract high-level attributes from image data and Convolutional Long Short-Term Memory (ConvLSTM) for capturing significant patterns from multisensory data. The extracted features from different modalities are then fused through self-attention mechanisms, which enhance relevant activity data and inhibit superfluous and possibly confusing information by measuring their compatibility. The proposed fusion architecture and two baseline architectures (CNN and ConvLSTM) are evaluated on the UP-Fall detection dataset, which consists of a sizeable multimodal benchmark. The fusion approach demonstrates superior performance compared to the baselines, achieving an accuracy of 97.61%, outperforming other state-of-the-art methods in the HAR literature.

Alexiadis et al. [93] presented a sensor-independent fusion method that allows for the design of multimodal methods operating with varying sensors, even when some sensor data are missing. To address the issue of missing sensor data and improve the fusion model’s accuracy, the authors proposed a data augmentation method that creates new observations using all possible subsets of activated sensors. The proposed methods were tested on the ExtraSensory dataset, which contains over 300,000 samples from 60 users and incorporates heterogeneous measurements from various wearable sensors, such as accelerometers, gyroscopes, magnetometers, watch compasses, and audio sensors. The dataset was used to fuse unimodal models for the available sensors. The results demonstrated that the sensor-independent fusion method enables the development of fusion models that can operate with fewer data sources than originally intended, as long as the maximum number of sensors is known beforehand.

Dhekane et al. [94] addressed the challenge of HAR in unannotated data streams generated by real-world smart home applications. In this context, they propose a real-time annotation framework for HAR based on Change Point Detection (CPD) and develop a novel transfer learning-based CPD algorithm called S-CPD. The algorithm calculates a Change Point Index (CPI) for each sensor reading in the data stream using similarities of output probability distributions, allowing for enhanced annotations. The authors emphasize the challenges posed by the multimodal nature of ubiquitous sensor data, which is heterogeneous and often noisy. Incorporating information from different sensor modalities into a single framework remains a prominent challenge in sensor-based HAR.

Hiremath et al. [95] presented a novel approach to boot-strapping HAR systems in smart homes. The authors acknowledge that starting an activity recognition system for specific smart homes is challenging due to the highly individualized nature of these spaces and the inhabitants’ unique behaviors. The proposed approach operates in a cold-start scenario, where the HAR system passively observes raw sensor data in the smart home without prior knowledge about the environment or its inhabitants. It then learns representations called action units, which are aggregated into activity models through a motif learning and discovery process that requires minimal supervision. The final HAR system can then recognize relevant and frequent activities in the smart home. The authors used an Active Learning-like procedure to minimize user burden during bootstrapping. Active learning provides annotations for a limited number of relevant and informative data points, reducing the need for large amounts of labeled data. This method is particularly useful in smart homes, where annotating large volumes of data can be time consuming and expensive. One potential application of the knowledge gained from the bootstrapping procedure is the utilization of additional sensor modalities. The authors suggest that the discovered knowledge about movement patterns and subsequences could be used to fine-tune HAR systems for specific tasks and assist smart home residents in their Activities of Daily Living (ADLs).

The works discussed in this section are summarized in Table 3.

Now that we have a grasp on how multimodality enhances the quality of data collected, let us delve into real-time processing. This step is critical in transforming the raw, multimodal data into useful information, providing timely and responsive services in smart living applications.

### 5.3. Real-Time Processing

In the realm of smart living applications, real-time processing plays a critical role in ensuring the seamless integration of technology into our daily lives. By enabling immediate analysis and response to various sensor inputs, real-time processing facilitates a smooth and intuitive user experience, ultimately improving the overall quality of life. Furthermore, real-time data processing is crucial for applications such as smart homes, healthcare monitoring, and security systems [96]. It allows for timely decision making, proactive intervention, and seamless adaptation to changing conditions.

To achieve real-time processing, developers can explore various approaches, including the choice of sensing modality, the implementation of lightweight computational frameworks, and other optimization techniques [97,98]. One approach to achieve real-time processing is by selecting an appropriate sensing modality. For example, video stream processing is computationally expensive, but utilizing depth cameras can help mitigate this issue. Depth cameras offer several advantages, such as easing segmentation algorithms, increased independence from lighting conditions, reducing noise, and providing richer spatial information [99].

Zin et al. [10] presented a real-time action recognition system for older adults using a stereo-depth camera. The system localizes people by extracting different regions of interest from UV-disparity maps. The experimental results demonstrate that the proposed system can detect various actions in real time with reasonable recognition rates, regardless of the length of the image sequences.

Wearable sensing is an additional modality that reduces processing load. While the complexity of wearable sensor data is typically lower than that of RGB cameras, their proximity to the body enables extensive data gathering, which makes them highly appropriate for HAR [100]. Another way to pursue real-time processing is by employing lightweight computational frameworks. These frameworks are designed with fewer parameters, low memory occupancy, and faster processing speeds, all contributing to lessening the computational load. Examples of such frameworks include MobileNet [76], SqueezeNet [101], and ShuffleNet [102], which deliver high performance while maintaining low resource requirements. Additionally, implementing model quantization and pruning techniques can further optimize these architectures, leading to more efficient processing.

Hu et al. [103] addressed the challenge of real-time activity recognition in health smart homes, focusing on optimizing SVMs (SVM) using genetic algorithms. The authors propose an online real-time activity recognition approach based on the genetic algorithm-optimized SVM classifier. The core of this approach involves a sliding window-based feature representation technique enhanced by the mutual information between sensors, which supports online real-time activity recognition. The authors’ proposed solution has two main aspects. Firstly, they design a sliding window-based feature extraction method that effectively reduces the influence of irrelevant information in a time window of sensor events by incorporating sensor mutual information into the feature vector. Secondly, they develop a multiclass SVM classification framework based on the feature mentioned above extraction technique for online real-time activity recognition.

In the paper by Chen et al. [104], the authors proposed a novel approach to HAR by utilizing skeleton extraction and image reconstruction. Traditional methods for HAR directly input source images, which can be affected by various factors such as heights, weights, poses, angles, and occlusions. In contrast, the proposed method uses the OpenPose library to extract 2D positions of human skeleton joints as a preprocessing phase. The skeleton image is then reconstructed from these joints, with coloring used to encode the categories of different human parts. These reconstructed images are input into a CNN structure for classification.

In their excellent work, Yan et al. [105] addressed the challenge of accurate and efficient real-time HAR in smart IoT systems by proposing a method that integrates offline and online learning to improve the performance of event-count sliding window techniques on streaming data. The authors use unsupervised learning to learn latent knowledge from explicit-activity window sequences, which helps to enhance the limited information of sliding windows without much domain knowledge. They then derive the probability distribution prediction of activity classes for a given sliding window. The researchers employ two unsupervised feature learning techniques, the enhanced topic-aware Bayesian approach, and the Hidden Markov Model (HMM)-based prediction, to consider activity classes within a window as the latent semantics or states underlying window feature/observation. They then feed the basic feature representation of a sliding window and the high-level feature representation, which is the probability distribution prediction of activity classes, into a classifier model to produce the activity class report for the window. The online activity report is produced once per event by processing the sliding window, which ends on that event.

Ramos et al. [106] presented a real-time human activity recognition system for monitoring the daily activities of elderly individuals. The system is developed using a prediction model based on bidirectional LSTM networks, which allows it to recognize real-time activities, a crucial feature for timely intervention in case of anomalies. The model is trained using data from the public CASAS dataset, which contains information from non-intrusive sensors installed in a person’s home. The authors employed data processing and filtering techniques, such as a sliding window method and a stacking and reordering algorithm, to ensure real-time processing and maintain a high accuracy rate. These methods enable the model to handle activities of varying durations and consider the time reference of each activity. The developed model provides an impressive 95.42% accuracy rate, outperforming existing models. One of the main strengths of this system is its ability to make real-time predictions with equispaced time intervals, addressing a limitation in previous approaches that required knowledge of the activity duration for making predictions.

The works discussed in this section are summarized in Table 4.

After understanding the significance of real-time processing, our attention turns towards interoperability. This domain is crucial for allowing seamless communication and data sharing across various devices and systems, enhancing the functionality and versatility of smart living environments.

### 5.4. Interoperability

Interoperability, a key aspect of any modern system, refers to the ability of different systems or components to work together in a coordinated manner, exchanging and utilizing information seamlessly. In the context of HAR in smart living, interoperability is crucial, as it enables integration with various smart home systems to provide users with a comprehensive and cohesive experience [107]. Interoperability is essential because it allows organizations to utilize different systems and technologies, saving time and money. Moreover, achieving semantic interoperability ensures that the real meaning of shared data is preserved across systems, applications, and devices, regardless of the vendor. This concept is particularly relevant in healthcare, where data sharing between clinicians, labs, hospitals, and pharmacies is vital for effective patient care.

In the context of HAR within smart living environments, interoperability refers to the seamless communication, interaction, and data exchange among different systems, devices, and protocols. More specifically, the following four levels of interoperability emerge from the analysis of the recent literature: (1) device and protocol interoperability, (2) data format interoperability, (3) semantic interoperability, and (4) interoperability in machine learning algorithms.

#### 5.4.1. Device and Protocol Interoperability

Enhancing interoperability among various devices and components in IoT systems is critical in realizing effective HAR in smart living environments, allowing for seamless communication and data exchange between connected appliances and sensors. This involves different devices (e.g., wearable sensors, ambient sensors, IoT appliances) and diverse networks or protocols (such as Wi-Fi, Zigbee, Bluetooth) working together. Zhang et al. [108] and Franco et al. [109] addressed this aspect with a layered IoT architecture and multiagent collaboration in smart homes.

Zhang et al. [108] proposed a knowledge-based approach for multiagent collaboration in smart homes, addressing issues such as device heterogeneity, composite activities recognition, and providing appropriate services. The authors developed a layered architecture of smart homes that combines ontology and multiagent technologies to automatically acquire semantic knowledge and support heterogeneity and interoperability services. This architecture is composed of four layers: the physical layer, the middleware layer, the knowledge management layer, and the service layer.

Franco et al. [109] proposed an IoT-based approach for load monitoring and activity recognition in smart homes, focusing on Intrusive Load Monitoring (ILM) techniques. The authors developed an IoT architecture composed of five layers: appliances, perception, communication network, middleware, and application. As part of the application layer, the appliance recognition module is responsible for labeling sensor data to enable the implementation of different home applications, such as activity recognition.

#### 5.4.2. Data Format Interoperability

Different devices generate data in various formats. For these devices to work together, their data needs to be understandable to each other. One way to achieve interoperability in such systems is by employing ontologies, which formally represent knowledge and facilitate data sharing among devices. By leveraging ontology-based approaches, fusing data from multiple sensing sources becomes possible, enhancing activity recognition systems’ performance and promoting seamless interoperability.

Noor et al. [110] used an ontology-based approach to fuse data from wearable and ambient sensors, ensuring data consistency across devices for recognizing ADLs in a smart home setting. The study aimed to resolve uncertainties due to missing sensor data by exploiting the advantages of both types of sensing. The authors proposed an ontology-based sensor fusion approach that combines user context provided by wearable sensor-based activity recognition with environmental contexts to handle missing sensor data.

#### 5.4.3. Semantic Interoperability

The capability of diverse systems to not only exchange information but also understand it at a higher level can be enhanced through the use of semantic fusion techniques. These techniques amalgamate data from a variety of sensors, leading to a comprehensive understanding of human activities in pervasive environments. Essentially, this means that these different systems can decipher the data—such as recognizing activities from sensor data—instead of merely transmitting it.

Stavropoulos et al. [72] used semantic fusion techniques, integrating computer vision and ambient sensors for activity recognition, ensuring the meaningful exchange of information. They presented a framework that integrates heterogeneous sensors for activity recognition in pervasive environments, specifically targeting the application of dementia care. The paper proposed a combination of ambient intelligence, semantic web technologies, computer vision, and Ambient Assisted Living (AAL) to provide real-world healthcare solutions. This integration addressed challenges for realistic applications, such as fast, efficient image analysis and ontology-based temporal interpretation models. The authors employed an ontology-based knowledge representation and semantic fusion for activity recognition using OWL as the underlying knowledge representation and reasoning language. The framework architecture included a sensor, analysis, representation, interpretation, service, and application layers, which provided a multidisciplinary approach for integrating various sensors and computer vision techniques.

#### 5.4.4. Interoperability in Machine Learning Algorithms

Various ML algorithms can be used for processing sensor data and recognizing human activities. Mekruksavanich et al. [111] and Minarno et al. [112] achieved interoperability by integrating different ML algorithms with sensor data for effective activity recognition.

Mekruksavanich et al. [111] proposed a framework for Exercise Activity Recognition (EAR) using Surface Electromyography (sEMG) data. Their approach is designed to improve recognition accuracy in applications such as AAL, smart healthcare, and smart rehabilitation. Interoperability is essential to their research, as it enables integrating sEMG data with various ML algorithms to recognize different exercise activities.

In their paper, Minarno et al. [112] explored the performance of various ML algorithms for activity recognition using accelerometer and gyroscope sensor data. Focusing on the interoperability aspect, the authors emphasize the relevance of autonomous systems in various applications, such as fall detection, medical rehabilitation, and smart home systems. Their system aims to significantly improve the quality of life by analyzing human physical activities, categorized into three classes: static, transition, and dynamic.

The works discussed in this section are summarized in Table 5.

With a clear picture of how interoperability contributes to the integration of various systems, let us move on to discuss resource-constrained processing. This domain is particularly important, as it addresses the inherent limitations in processing power, energy, and memory in many smart living devices, thereby optimizing the performance and sustainability of HAR systems.

### 5.5. Resource-Constrained Processing

In the context of smart living and HAR applications, addressing the challenges posed by limited processing resources is essential. These constraints arise due to the demand for affordable, energy-efficient devices seamlessly integrating into home environments. Such devices often rely on mobile or wireless platforms, presenting limitations such as restricted processing power, storage, bandwidth, and power resources. To tackle the issue of limited processing power, low-cost and energy-efficient devices should employ lightweight algorithms, which are both computationally efficient and effective at recognizing human actions. These algorithms should be optimized for mobile or wireless platforms, ensuring the best performance on such devices [114].

Regarding limited storage resources, utilizing efficient algorithms and data structures to handle large volumes of data generated by HAR applications is crucial. This may involve implementing innovative techniques such as data pruning or feature selection, which help reduce the dataset’s size while preserving its essential information. By doing so, devices can store and process the data effectively, even with limited storage capacity. Addressing limited bandwidth for data transmission is another challenge in smart living and HAR applications. To overcome this hurdle, developing efficient compression and communication techniques is crucial. These methods should minimize the amount of data transmitted between devices while maintaining the quality of shared information. This can be achieved through the use of advanced data compression techniques, as well as optimized protocols for data communication [115].

Moreover, concerning limited power resources, it is important to design efficient algorithms and power management techniques that ensure long battery life for portable devices. This may involve dynamic power management strategies, such as adaptive duty cycling, which adjusts the device’s power consumption based on its current workload. In addition, energy-aware algorithms can be employed in HAR applications to minimize the power consumption of the devices, prolonging their battery life without compromising their performance.

Zhou et al. [116] proposed an innovative HAR system based on Improved Bayesian Convolution Network (IBCN) for processing on limited-resource devices. The study’s primary focus was addressing resource-constrained processing in Wearable Internet of Things (W-IoT) devices used for data analysis.

The IBCN approach allows each smart system to download data using either traditional RF or low-power back-dispersion communication with cloud assistance. The authors designed a distribution of the model’s latent variable and extracted features using convolution layers, aiming to improve the performance of W-IoT devices. Combining a variable autoencoder with a standard deep net classifier was used to achieve this goal.

In their research, Chang et al. [117] focused on developing a low-power, memory-efficient, and high-speed ML algorithm for smart home activity data classification suitable for resource-constrained environments. However, considering the numerous publicly available HAR datasets, the authors’ decision to use the MNIST dataset [118] as a substitute for real-world activity data is questionable. While they argue that experimental constraints and similarities between datasets when converted to image form justify their choice, whether the MNIST dataset adequately represents human activity data’s complexity and unique features are debatable. The proposed ML algorithm consists of data preprocessing, training, and classification stages. In data preprocessing, training data with the same label are grouped into detailed clusters. The classification algorithm is based on an enhanced SVM, in which the training process generates hyperplanes by accumulating and thresholding from each cluster of preprocessed data. The classification process classifies input data by calculating the similarity between the input data and each hyperplane using a bitwise-operation-based error function.

The authors implemented the proposed algorithm on Raspberry Pi 3 and STM32 Discovery board embedded systems. The authors noted that although the proposed model had fast execution time and efficient memory and power usage, the model’s accuracy and other evaluation metrics were slightly lower than conventional ML/DL approaches. More research is needed to prove the suitability of using the MNIST dataset to represent real-world activities. Future work should optimize the model for real-world activity data to achieve better accuracy while preserving efficient resource consumption.

The paper by Zhu et al. [119] proposed a lightweight CNN architecture named Mobile-RadarNet, for HAR based on micro-Doppler signatures in resource-constrained mobile-edge computing devices. The authors address the issue of computational complexity and model size, which limit the deployment of DL models in mobile devices. The proposed architecture uses 1D depthwise and pointwise convolutions to build a streamlined and efficient network, which extracts features in each frequency bin and exchanges information between frequency bins. The authors show that the proposed Mobile-RadarNet achieves high classification accuracy while keeping the computational complexity shallow, making it suitable for mobile device deployment.

The paper by Helmi et al. [120] proposed a new method for HAR using wearable sensor data. The method integrates DL and swarm intelligence optimization algorithms to build a robust HAR system that addresses the feature selection problem. The authors developed a light feature extraction approach using the Residual Convolutional Network (RCN) and an RNN and proposed three variants of the Marine Predator Algorithm (MPA) for feature selection. The MPA variants use two widely used transfer functions to perform the binarization process, including the V-shaped transfer function for MPA (MPAV). The study employs three public datasets containing complex and comprehensive activities: Opportunity, PAMAP2, and UniMiB-SHAR. It compares the proposed MPA variants to several optimization algorithms using two classifiers, i.e., the SVM classifier and the Random Forest Classifier (RFC). The evaluation experiments show that the MPAV recorded the best performance compared to other MPA variants and other compared methods.

The authors compare HAR models based on complexity and resource usage in the paper by Angerbauer et al. [121]. The study compares traditional ML models, such as Random Forest Classifier (RFC) and SVM, with more complex DL models, namely CNNs and RNNs. The authors assess the complexity of the models by considering memory consumption, mean prediction time, and the number of trainable parameters. All models are tested on the same publicly available UCI-HAR Smartphone dataset to ensure a fair comparison. The results show that while the DL models perform similarly to the traditional ML models in recognition accuracy, their increased complexity renders them less favorable for practical applications. The RF model is considered the best option for memory-limited use cases, with an F1-Score of 88.34%, memory consumption of only 0.1 MB, and a mean prediction time of 0.22 ms. The overall best model in complexity and performance is the SVM with a linear kernel, achieving an F1-Score of 95.62%, memory consumption of 2 MB, and a mean prediction time of 0.47 ms.

In HAR, smartphones have become a vital tool due to their embedded accelerometers and gyroscopes. These sensors can monitor daily activities, such as elderly care, healthcare, sports, and smart homes. However, the high-dimensional feature vectors generated from smartphone sensor data pose challenges in processing and classification, leading to the “curse of dimensionality” phenomenon. Ahmed et al. [122] addressed this issue by proposing a hybrid feature selection model that combines filter and wrapper methods to achieve efficient HAR in resource-constrained environments. The authors employed a Sequential Floating Forward Search (SFFS) technique to extract relevant features fed into a multiclass SVM to create nonlinear classifiers. The proposed system demonstrates satisfactory activity identification even on limited hardware resources by adopting the kernel trick for training and testing purposes. The significance of this research lies in its ability to provide enhanced HAR performance while mitigating the challenges posed by high-dimensional feature vectors. The hybrid feature selection model proposed by the authors enables the development of efficient and accurate HAR systems suitable for applications in various aspects of daily life, with a particular focus on resource-constrained processing.

The works discussed in this section are summarized in Table 6.

After exploring the challenges and strategies associated with resource-constrained processing, we are now ready to engage in a critical discussion. Here, we will synthesize the insights gained from each domain and reflect on the broader implications for the development of effective and sustainable HAR systems for smart living applications.

## 6. Critical Discussion

The analytical framework suggested in this study provides a comprehensive perspective on the main domains of HAR in smart living. However, it is crucial to analyze these domains critically to ensure that the development of smart living environments addresses potential concerns and challenges.

### 6.1. Sensing Technologies

The critical discussion on sensing technologies reveals the importance of considering not only the individual capabilities of these technologies, but also their potential synergies and interactions within a broader HAR system. Developing more effective and user-friendly smart living solutions requires taking a holistic approach that accounts for the diverse range of available sensing technologies and their associated strengths and limitations.

A crucial aspect that emerges from the discussion is the need to balance the trade-offs between various factors, such as accuracy, computational resources, power consumption, and user acceptability. Each sensing technology comes with its unique set of advantages and challenges, necessitating a careful evaluation of their suitability for specific applications and contexts. For instance, while high-accuracy sensors might provide rich contextual information, they may also demand more computational power or energy, making them less suitable for certain applications.

One of the key considerations when selecting sensing technologies for HAR systems is their perceived intrusiveness. To ensure user acceptance and adoption, it is essential to address privacy concerns and minimize the invasiveness of the technologies. For example, solutions such as RF sensing offer the advantage of non-intrusive monitoring without the need for cameras or wearable devices, making them more appealing to users. However, further research and development is required to enhance the capabilities of these technologies in detecting more complex actions and providing a comprehensive understanding of human activities. In addition, it is important to note that the use of RF sensing also raises concerns regarding Electromagnetic Interference (EMI) in the presence of medical devices such as pacemakers and implanted defibrillators.

Another important aspect is the development of sensor fusion techniques that can effectively combine data from multiple sensing modalities to provide a more accurate and robust HAR system. By leveraging the complementary strengths of different sensing technologies, researchers can address the limitations of individual sensors and enhance the overall performance of the system. Sensor fusion can also lead to more adaptive and context-aware solutions that can cater to the diverse needs of users and their environments.

In addition to focusing on the technical aspects of sensing technologies, it is also essential to consider the broader societal implications of their widespread adoption in smart living environments. The establishment of ethical guidelines and regulations is crucial to ensuring the responsible use of these technologies and protecting users’ rights. As sensing technologies continue to advance and become more pervasive, it is essential to engage in open dialogues and interdisciplinary collaborations that address the ethical, legal, and social implications of their deployment.

### 6.2. Multimodality

The increased complexity resulting from the integration of multiple sensing modalities can lead to resource-intensive management. This is evident in the development of DL models, such as CNNs, LSTM networks, and transformer networks, that need to effectively model spatial-temporal dependencies of sensor signals and address the distinct contributions of different sensing modalities in complex environments. Researchers have proposed various solutions, such as self-attention-based models and two-stream structures, to tackle these challenges. However, the resource-intensive nature of these solutions may limit their applicability in certain scenarios.

Furthermore, incorporating multiple sensors in multimodal sensing could raise privacy concerns as more user activity data are collected. For example, the comprehensive multimodal dataset presented by Bocus et al. [24] includes synchronized RF devices, WiFi signals, UWB signals, and vision/infrared data from Kinect sensors. While such datasets accelerate the development of self-supervised learning techniques, they also highlight the need to balance data collection with users’ privacy rights.

The heterogeneity of data collected from various sensors presents additional challenges in multimodal HAR. This heterogeneity may result in missing sensor data or noisy and unreliable measurements. To overcome these challenges, researchers have proposed data augmentation methods, sensor-independent fusion techniques, and transfer learning-based algorithms. However, the practical implementation of these approaches may require further refinements to ensure robustness and adaptability across different real-world scenarios.

Finally, Bootstrapping HAR systems in smart homes can be particularly challenging due to the highly individualized nature of these environments and the unique behaviors of inhabitants. To minimize user burden and reduce the need for large amounts of labeled data, researchers have proposed active learning-like procedures [95]. These methods may help discover knowledge about movement patterns and subsequences to fine-tune HAR systems for specific tasks. However, the effectiveness of these approaches in diverse and complex smart home settings remains an open question.

### 6.3. Real-Time Processing

The critical discussion of real-time processing in smart living applications showcases the need for a balance between processing efficiency, accuracy, and the choice of sensing modalities. Drawing from this analysis, we can derive several general indications that are applicable beyond the specific reviewed works. The choice of sensing modality significantly influences the system’s ability to achieve real-time processing. It is essential to select the appropriate sensing technology based on the requirements of the specific application. While certain modalities, such as depth cameras or wearable sensing, can reduce computational complexity and facilitate real-time processing, their effectiveness may vary depending on the environment, type of activities, and data quality requirements. Thus, a thorough assessment of the application’s goals and constraints should guide the selection of sensing modalities to ensure the desired balance between processing speed and data quality.

Leveraging lightweight computational models and optimization techniques is crucial for enabling real-time processing in smart living applications. By employing models with fewer parameters, lower memory occupancy, and faster processing speeds, developers can ensure efficient analysis and response to sensor inputs. Optimization techniques, such as model quantization and pruning, can further enhance processing efficiency while maintaining acceptable levels of accuracy. However, the choice of lightweight models should consider the potential trade-offs in terms of performance and the ability to learn complex representations. In cases where high accuracy and complex modeling are essential, researchers may need to explore more advanced optimization techniques.

Incorporating data preprocessing and feature extraction and selection techniques can significantly improve the efficiency and accuracy of real-time processing [10,105]. Techniques such as sliding window-based feature representation, skeleton extraction, and image reconstruction can help reduce the influence of irrelevant information, enhance data quality, and simplify the input to the models. These methods can lead to improved recognition accuracy and faster processing times. Nevertheless, developers should carefully consider the limitations of these techniques, such as sensitivity to data quality or extraction errors, and evaluate their suitability for the target application.

Real-time processing systems should prioritize user privacy and security, especially in applications involving monitoring and decision making. The use of non-intrusive sensors and privacy-preserving techniques can help maintain user autonomy and trust in the system. It is vital to ensure that the pursuit of real-time processing does not compromise the privacy and security requirements of the application.

Adaptability and scalability are essential considerations for real-time processing systems. As smart living applications evolve and new technologies emerge, the ability to adapt and scale the system becomes increasingly important. Developers should design systems with the flexibility to incorporate new sensing modalities, computational models, and optimization techniques. This adaptability ensures that the system remains effective and efficient in handling real-time processing tasks as the application requirements and technological landscape evolve.

### 6.4. Interoperability

It is evident that standardization is crucial for achieving interoperability. With a multitude of devices and systems from different vendors in the market, the lack of common standards can hinder seamless integration and communication. Standardization not only facilitates data exchange, but also ensures the compatibility of systems, making it easier for organizations to adopt and implement new technologies. Therefore, it is necessary for stakeholders, including manufacturers, developers, and researchers, to collaborate and develop open standards that promote interoperability.

The use of ontologies and semantic technologies can greatly enhance interoperability across various domains. Ontologies provide a formal representation of knowledge and facilitate the sharing and understanding of data among devices and systems. By adopting ontology-based approaches, organizations can promote the seamless fusion of data from multiple sources, thereby enhancing the overall performance of their systems. Semantic technologies also enable the preservation of the real meaning of shared data across systems, applications, and devices, which is particularly important in fields such as healthcare and AAL, where accurate and meaningful data sharing is essential.

Achieving interoperability may require continuous updates and adaptation. As technology evolves and new devices are introduced, systems need to be updated and adapted to maintain accuracy and effectiveness. This may involve retraining classifiers with new data or updating algorithms to accommodate changes in the environment. Organizations should be prepared to invest in the necessary resources and efforts to ensure that their systems remain interoperable and up to date.

The integration of various data sources and sensing modalities can lead to more accurate and efficient systems. Interoperability allows for the seamless exchange of information between wearable and ambient sensors, resulting in a comprehensive understanding of human activities and events. This can be particularly beneficial in fields such as healthcare, where the monitoring and recognition of activities are crucial for providing effective patient care. By leveraging diverse sensing technologies and promoting interoperability, organizations can develop more accurate and robust systems that cater to the diverse needs of their users.

Lastly, interoperability fosters innovation and collaboration. When systems and devices can communicate and exchange data seamlessly, it opens new possibilities for the development of innovative solutions that address complex challenges in various domains. By prioritizing interoperability, stakeholders can work together to create more versatile, user-friendly, and adaptive solutions that can be easily integrated into different environments and cater to the diverse needs of users.

### 6.5. Resource-Constrained Processing

One crucial consideration when dealing with resource-constrained processing is the need for computational efficiency. This is applicable to various areas, such as healthcare, environmental monitoring, and industrial automation, where devices must perform complex tasks with limited resources [115,120]. The use of lightweight algorithms and architectures that minimize computational overhead can be instrumental in addressing these challenges. However, it is essential to maintain a balance between computational efficiency and the desired level of accuracy and reliability. Ongoing research and innovation in algorithm design and optimization can help achieve this balance, ensuring that the resulting solutions are both efficient and effective.

Data management is another critical aspect of resource-constrained processing. In many application domains, vast amounts of data are generated, collected, and processed, leading to storage and transmission challenges. Efficient algorithms and data structures for handling large volumes of data can be invaluable in mitigating these issues. Techniques such as data pruning, feature selection, and data compression can be employed to reduce dataset size while preserving essential information. However, the trade-off between data reduction and information integrity must be carefully managed. Additionally, optimizing communication protocols to handle limited bandwidth and ensure reliable data transmission is vital.

Energy efficiency is a fundamental concern in resource-constrained environments, particularly in portable and battery-powered devices. Designing efficient algorithms and power management techniques that ensure long battery life without compromising performance is essential. Dynamic power management strategies and energy-aware algorithms can help strike a balance between energy consumption and device performance. However, continuous research and innovation are necessary to adapt these strategies to varying workloads and energy demands. Moreover, incorporating energy-harvesting technologies and exploring alternative energy sources could also contribute to more sustainable and energy-efficient solutions.

Addressing resource-constrained processing requires a holistic approach that encompasses computational efficiency, data management, and energy efficiency. By continuously researching and innovating in algorithm design, data handling techniques, communication protocols, x and energy management strategies, researchers and developers can contribute to more efficient, accessible, and sustainable solutions that cater to various users and environments. To achieve this goal, interdisciplinary collaboration, including computer science, engineering, and domain-specific expertise, is necessary to ensure that the resulting solutions meet the diverse needs and constraints of real-world applications. Additionally, fostering a research culture that prioritizes resource-constrained processing and shares best practices across different domains can help accelerate progress and pave the way for more effective and sustainable technology solutions.

A pivotal aspect of dealing with resource-constrained processing is the pursuit of computational efficiency, particularly critical in fields such as healthcare, environmental monitoring, and industrial automation. In these areas, devices are often tasked with executing complex operations within stringent resource limitations [115,120]. Harnessing lightweight algorithms and streamlined architectures that reduce computational overhead is a key strategy in navigating these limitations. Nevertheless, striking a balanced approach is vital, as there is a delicate trade-off between computational efficiency and achieving the necessary degree of accuracy and reliability. Real-time applications necessitate this balance as they often involve continuous monitoring and real-time decision making. Ensuring that ongoing research in algorithmic design and optimization acknowledges this trade-off is pivotal in crafting solutions that are both efficient and robust.

### 6.6. A Short Note on Sensing Modalities

The type of sensor used for HAR significantly impacts the range of detectable human actions. It is important to note that no single sensing modality can address all HAR’s challenges. Each type of sensor has its strengths and limitations, and selecting the appropriate sensors depends on the specific application and the system’s objectives. For instance, while cameras offer a high level of detail and can recognize intricate gestures and facial expressions, they may not be the best choice for applications that require privacy protection.

Similarly, while wearable sensors can detect basic activities such as walking or running, they may not be suitable for applications that require the recognition of fine-grained gestures or facial expressions. Therefore, a combination of sensors with different modalities may be required to address the various challenges of HAR. When designing the system, researchers should consider the trade-offs between accuracy, privacy, intrusiveness, and cost. Given these challenges, researchers should consider adopting a multimodal approach, combining data from different sensor types to leverage their strengths and mitigate their shortcomings.

### 6.7. A Short Note on Wearable Solutions

Positioning sensors on the body plays a critical role in the effectiveness of wearable solutions for HAR. While some studies focus on using a single device placed on the waist or shoulders for low intrusiveness, this approach limits the range of detectable actions to coarse movements, such as walking or sitting. This limitation makes it difficult to recognize more complex activities or subtle gestures that may be relevant to the overall system design.

To overcome this challenge, researchers could explore using multiple sensors strategically placed on various body parts, such as wrists, ankles, or the head [82,90,97]. This approach can significantly improve the range of detectable actions and enable the recognition of fine-grained gestures, such as hand movements or facial expressions. However, this approach may introduce additional complexity to the system design, such as synchronizing data streams from multiple sensors. Moreover, using multiple sensors can lead to a more intrusive solution, which may not be desirable for some users.

Alternatively, researchers could investigate using advanced algorithms that can extract more information from a single sensor, enabling the detection of complex actions even with limited sensor data. This approach can minimize the number of sensors needed, reducing the system’s complexity, cost, and intrusiveness. However, developing such advanced algorithms may require significant computational resources and focus on ML and signal processing techniques. Furthermore, the choice between using multiple sensors or advanced algorithms for HAR depends on factors such as the system’s specific application, cost, power consumption, and processing capabilities. Researchers must carefully evaluate the trade-offs between these factors to develop effective and practical solutions for wearable HAR.

## 7. The Authors’ Perspective

This review paper, offering an updated state-of-the-art examination of HAR for smart living environments, was crafted within the context of the 4FRAILTY project. As researchers deeply embedded within this project, our involvement shaped the review process, lending our insights and experiences to the broader overview of the field.

4FRAILTY is an ambitious project dedicated to developing an integrated architecture for living environments to improve the quality of life for frail individuals residing in their homes. Central to the project is a telemonitoring system that leverages wearable technologies and smart devices to keep the patients within a defined “safety zone”. This system works with a Decision Support System to aid caregivers in the early detection of risks and implementing timely, low-cost interventions.

One of the essential characteristics of the project is its innovative focus, developing non-invasive, affordable technologies that are efficient and reliable. By deploying advanced solutions in life environments through the utilization of microelectronics, sensors, new materials, and IoT, the project targets some of the leading Key Enabling Technologies (KETs) that will shape the future of this field.

## 8. Conclusions

This exhaustive review has presented a rigorous and comprehensive exploration of the critical domains involved in HAR for Smart Living applications. The study has been meticulously organized and carried out with a focus on five crucial dimensions: Sensing Technology, Multimodality, Real-time Processing, Interoperability, and Resource-Constrained Processing.

Throughout this study, a robust body of scholarly work has been examined, with a critical analysis carried out on more than 100 papers from the last two years, carefully chosen from a pool of 511 documents. This rigorous methodology has allowed for an in-depth understanding of the cutting-edge developments in the field, paving the way for the formulation of key insights and lessons learned across all dimensions.

This comprehensive analysis and the consequent synthesis of key insights and lessons are instrumental in understanding the current landscape of HAR in Smart Living environments, identifying the challenges that need to be addressed, and highlighting potential pathways for future research and innovation. This review, thus, contributes significantly to the ongoing scholarly discourse in the field and serves as a valuable resource for researchers, developers, and practitioners alike.

The discussion on sensing technologies for HAR systems highlights several key insights and lessons:

- Holistic Approach to Sensing Technologies: Smart living solutions need a comprehensive view considering various sensing technologies, their strengths, limitations, and potential synergies in a HAR system.
- Trade-offs between Multiple Factors: Sensing technologies require balancing accuracy, computational resources, power use, and user acceptability. Tailored solutions often outperform a one-size-fits-all approach in HAR systems.
- Perceived Intrusiveness and Privacy Concerns: HAR’s success depends on user acceptance, necessitating minimization of invasiveness and addressing privacy issues. RF sensing is less intrusive but not without concerns, similar to EMI.
- Sensor Fusion: Techniques to combine data from multiple sensors can improve HAR system accuracy and robustness, leveraging the strengths of different sensing technologies to overcome individual limitations.
- Societal Implications: Beyond technical aspects, sensing technology adoption has broader societal implications. Interdisciplinary collaborations and open dialogues are needed to address ethical, legal, and social consequences, emphasizing the need for ethical guidelines and regulations.

The discussion of HAR systems focusing on multiple sensing modalities highlights several complex issues and important lessons:

- Resource Intensity: Handling multiple sensing modalities in HAR systems is resource-intensive, and while there are proposed solutions, their resource demands may limit applicability.
- Privacy Concerns: Collecting large amounts of user data for multimodal sensing raises privacy issues that require careful handling.
- Data Heterogeneity: The diverse data from various sensors can lead to missing or unreliable measurements. Proposed solutions, such as data augmentation and sensor-independent fusion techniques, need further refinement.
- Individualized Environments and User Behaviors: Developing HAR systems often requires extensive knowledge of the environment and behaviors. Though feasible in laboratory conditions, this is impractical in real settings. State-of-the-art advancements generally prioritize recognition performance, overlooking the initial setup. Thus, approaches identifying activity sequences from motion patterns and incorporating higher-level functionality via bootstrapping could be beneficial.

The discussion on real-time processing in HAR systems reveals these lessons:

- Choice of Sensing Modality: The selection of sensing technologies greatly impacts the system’s real-time processing ability. Each modality, such as depth cameras or wearable sensors, has its pros and cons, and their effectiveness varies based on the environment, type of activities, and data quality requirements.
- Optimization of Computational Models: Lightweight computational models and optimization techniques, such as model quantization and pruning are essential for real-time processing. However, trade-offs between performance and the ability to learn complex representations need careful consideration.
- Data Preprocessing and Feature Extraction: Techniques such as sliding window-based feature representation and skeleton extraction can improve real-time processing efficiency and accuracy, but their limitations and suitability should be carefully evaluated.
- Privacy and Security: User privacy and security should not be compromised in the quest for real-time processing. The use of non-intrusive sensors and privacy-preserving techniques can help maintain user trust.
- Adaptability and Scalability: HAR systems should be adaptable and scalable to incorporate new sensing modalities, computational models, and optimization techniques as technology evolves.

Interoperability lessons from the discussion:

- Standardization: It is critical for seamless integration and communication among diverse devices and systems. Stakeholders must collaborate to develop open standards that promote interoperability.
- Ontologies and Semantic Technologies: They enhance interoperability by providing a formal representation of knowledge and facilitating meaningful data sharing across devices and systems.
- Continuous Updates and Adaptation: As technologies evolve, systems need ongoing updates and adaptations to maintain accuracy and effectiveness and to ensure interoperability. This requires investment in resources and effort.
- Integration of Data Sources: Interoperability enables seamless data exchange between different sensors, improving system accuracy and efficiency. This is particularly beneficial in healthcare and other domains where monitoring and recognition of activities are crucial.
- Innovation and Collaboration: Interoperability fosters innovative solutions that address complex challenges and can be integrated into diverse environments to cater to varying user needs.

The discussion on resource-constrained processing reveals the following lessons:

- Computational Efficiency: The balance between computational efficiency and accuracy is crucial, requiring lightweight algorithms and architectures. Yet, maintaining accuracy while reducing load remains a challenge.
- Data Management: Dealing with large amounts of data requires effective algorithms and techniques such as data pruning and compression. Maintaining a balance between reducing the data and preserving its integrity while also implementing efficient communication protocols for dependable data transmission is crucial.
- Energy Efficiency: Energy-efficient algorithms and dynamic power management are key, especially for portable devices. Exploring energy-harvesting technologies and alternative energy sources is an important future direction.
- Holistic Approach: Addressing resource constraints requires interdisciplinary collaboration and a culture of research prioritizing resource-constrained processing. Ongoing innovation is necessary for developing and refining effective and sustainable technology solutions.

While there have been notable advancements in HAR systems, a definitive, market-ready solution is yet to be realized. The primary reason is the delicate interplay between numerous factors and the highly personalized nature of smart living systems. The existing solutions often need to be revised, providing incomplete solutions to users’ needs because of these complexities. Hence, instead of chasing a one-size-fits-all solution, the current research and development efforts focus more on identifying and solving specific real-world problems, creating bespoke solutions for them. In conclusion, the field is moving towards customization, addressing unique issues with tailored responses.

Our ongoing and future work will involve further in-depth analysis of the extensive corpus of papers. This continued examination aims to delve deeper into the impact of smart living services and applications. We believe this further exploration will reveal subtler nuances and complexities in these domains and provide us with a more nuanced understanding of their implications for developing HAR systems. Additionally, we plan to extend our analysis to other dimensions not extensively covered in this review but equally critical in HAR systems. We aim to investigate areas such as Context Awareness, Personalization, Privacy, and Data Availability.

## Figures and Tables

**Table 1:** Common publicly available datasets.

Reference	Name	Sensors	Subjects No/Type of Environment	Actions/Contexts
Roggen et al. [42]	Opportunity	12/Lab	Body-worn, object-attached, ambient sensors (microphones, cameras, pressure sensors).	Getting up, grooming, relaxing, preparing/consuming coffee/sandwich, and cleaning up; Opening/closing doors, drawers, fridge, dishwasher, turning lights on/off, drinking.
Reiss and Stricker [43]	PAMAP2	9/Lab	IMUs, ECG.	Lie, sit, stand, walk, run, cycle, iron, vacuum clean, rope jump, ascend/descend stairs, watch TV, computer work, drive car, fold laundry, clean house, play soccer.
Micucci et al. [44]	UniMiB SHAR	30/Lab	Acceleration sensor in a Samsung Galaxy Nexus I9250 smartphone.	ADLs (Walking, Going upstairs/downstairs, Sitting down, Running, Standing up from sitting/laying, Lying down from standing, and Jumping, eight types of falls.
Cook and Diane [45]	CASAS: Aruba	1 adult, 2 occasional visitors/Real world	Environment sensors: motion, light, door, and temperature.	Movement from bed to bathroom, eating, getting/leaving home, housework, preparing food, relaxing, sleeping, washing dishes, working.
Cook et al. [46]	CASAS: Cairo	2 adults, 1 dog/Real world	Environment sensors: motion and light sensors.	Bed (four different types), bed to toilet, breakfast, dinner, laundry, leave home, lunch, night wandering, work, medicine.
Cook and Schmitter-Edgecombe [47]	CASAS: Kyoto Daily life	20/Real world	Environment sensors: motion, associated with objects, from the medicine box, a flowerpot, a diary, a closet, water, kitchen, and telephone use sensors.	Making a call, washing hands, cooking, eating, washing the dishes.
Singla et al. [48]	CASAS: Kyoto Multiresident	2 (pairs taken from 40 participants)/ Real world	Environment sensors: motion, item, cabinet, water, burner, phone and temperature.	Medication dispensing, clothes hanging, furniture moving, lounging, plant watering, kitchen sweeping, checkers playing, dinner prep, table setting, magazine reading, bill paying simulation, picnic prep, dishes retrieving, picnic packing.
Cook and Schmitter-Edgecombe [47]	CASAS: Milan	1 woman, 1 dog, 1 occasional visitor/ Real world	Environment sensors: motion, temperature, door closure.	Bathing, bed to toilet, cook, eat, leave home, read, watch TV, sleep, take medicine, work (desk, chores), meditation.
Cook et al. [49]	CASAS: Tokyo	2/Real world	Environment sensors: motion, door closure, light.	Working, preparing meals, and sleeping.
Cook and Schmitter-Edgecombe [47]	CASAS: Tulum	2/Real world	Environment sensors: motion, temperature.	Breakfast, lunch, enter home, group meeting, leave home, snack, wash dishes, watch TV.
Weiss et al. [50]	WISDM	51 (undergraduate and graduate university students between the ages of 18 and 25)/Lab	Accelerometer and gyroscope sensors, which are available in both smartphones and smartwatches.	Walking, jogging, stair-climbing, sitting, standing, soccer kicking, basketball dribbling, tennis catching, typing, writing, clapping, teeth brushing, clothes folding, eating (pasta, soup, sandwich, chips), cup drinking.
Vaizman et al. [51]	ExtraSensory	60/Real world	Accelerometers, gyroscopes, and magnetometers sensors, which are available in both smartphones and smartwatches.	Sitting, walking, lying, standing, bicycling, running outdoors, talking, exercise at the gym, drinking, watching TV, traveling on a bus while standing.
Zhang et al. [52]	USC-HAD	14/Lab	three-axis accelerometer, three-axis gyroscope, and a three-axis magnetometer.	Walking, walking upstairs/downstairs, running, jumping, sitting, standing, sleeping, elevator up/down.
Stiefmeier et al. [53]	Skoda	8/Lab	RFID tags and readers, force-sensitive resistors, IMUs (Xsens), ultrawideband position (Ubisense).	Opening the trunk, closing the engine hood, opening the back right door, checking the fuel, opening both right doors, check gaps at the front left door.
Martinez-Villasenor et al. [54]	UP-Fall	17/Lab	Accelerometer, gyroscope, ambient light sensor, electroencephalograph (EEG), infrared sensors, cameras.	Walking, standing, picking up an object, sitting, jumping, laying down, falling forward using hands, falling forward using knees, falling backward, falling sitting in an empty chair, falling sideward.
UK-DALE dataset [55]	UK-DALE	N.A./Real world	Smart plugs.	Energy consumption of appliances: dishwasher, electric oven, fridge, heat pump, tumble dryer, washing machine.
Arrotta et al. [56]	MARBLE	12/Lab	Inertial (smartwatch), magnetic, pressure, plug.	Answering phone, clearing table, cooking, eating, entering/leaving home, making phone call, preparing cold meal, setting up table, taking medicines, using PC, washing dishes, watching TV.
Schuldt et al. [57]	KTH	25/Lab	Cameras.	Walking, jogging, running, boxing, hand waving, hand clapping.
Blank et al. [58]	Weizmann	9/Lab	Cameras.	Running, walking, jumping-jack, jumping-forward-on-two-legs, jumping-in-place-on-two-legs, galloping-sideways, waving-two-hands, waving-one-hand, bending.
Rodriguez et al. [59]	UCF Sports Action	N.A./Lab	Cameras.	Sports-related actions.
Sucerquia et al. [60]	SisFall	38/Lab	Accelerometer and gyroscope fixed to the waist of the participants.	Walking/jogging falls (slips, trips, fainting), rising/sitting falls, sitting falls (fainting, sleep), walking (slow, quick), jogging (slow, quick), stair-climbing (slow, quick), chair sitting/standing (half, low height), chair collapsing, lying-sitting transitions, ground position changes, bending-standing, car entering/exiting, walking stumbling, gentle jumping.
Niemann et al. [61]	LARa	14/Lab	Optical marker-based Motion Capture (OMoCap), inertial measurement units (IMUs), and an RGB camera.	Standing, walking, using a cart, handling objects in different directions, synchronization.
Anguita et al. [62]	UCI-HAR	30/Lab	Accelerometers and gyroscopes embedded in a Samsung Galaxy S II smartphone.	Standing, sitting, laying down, walking, walking downstairs/upstairs.
Shoaib et al. [63]	UT_complex	10/Lab	Accelerometer, gyroscope, and linear acceleration sensor in a smartphone.	Walking, jogging, biking, walking upstairs/downstairs, sitting, standing, eating, typing, writing, drinking coffee, giving a talk, smoking one cigarette.
Chen et al. [64]	UTD-MHAD	8/Lab	Kinect camera and a wearable inertial sensor.	Sports actions (bowling, tennis serve, baseball swing), hand gestures (drawing ‘x’, triangle, circle), daily activities (door knocking, sit-stand, stand-sit transitions), training exercises (arm curl, lunge, squat).
Reyes-Ortiz et al. [65]	UCI-SBHAR	30/Lab	IMU of a smartphone carried out on the waist.	Standing, sitting, laying down, walking, walking downstairs/upstairs.

**Table 2:** Sensing technologies.

Reference	Methods	Dataset/s	Performance	Sensor/s	Actions
Ramos et al. [66]	DL techniques based on recurrent neural networks are used for activity recognition.	Self collected (SDHAR-HOME dataset), made public. Two users living in the same household.	A = 0.8829 (min.), A = 0.9091 (max).	Motion, Door, Window, Temperature, Humidity, Vibration, Smart Plug, Light Intensity.	Taking medication, preparing meals, personal hygiene
Arrotta et al. [67]	Combined semi-supervised learning and knowledge-based reasoning, cache-based active learning.	MARBLE	F1S = 0.89 (avg.).	Smartwatches equipped with inertial sensors, positioning system data, magnetic sensors, pressure mat sensors, and plug sensors.	Answering the phone, cooking, eating, washing dishes.
Khater et al. [68]	ConvLSTM, ResIncConvLSTM layer (residual and inception combined with ConvLSTM layer),	KTH, Weizmann, UCF Sports Action.	A = 0.695 (min.), A = 0.999 (max.).	Cameras.	Walking, jogging, running, boxing, dual hand waving, clapping, side galloping, bending, single hand waving, spot jumping, jumping jack, skipping, diving, golf swinging, kicking, lifting, horse riding, skateboarding, bench swinging, side swinging.
Mohtadifar et al. [69]	Doppler shift, Mel-spectrogram feature, feature-level fusion, six classifiers: MLP, SVM, RFC, ERT, KNN, and GTB.	Self collected involving four subjects.	A = 0.98 (avg.).	Radio frequency (Vector Network Analyzer), microphone array.	Falling, walking, sitting on a chair, standing up from a chair.
Delaine et al. [70]	New metrics for a priori estimation of the performance of model-based activity recognition: participation counter, sharing rate, weight, elementary contribution, and distinguishability.	Self collected involving one subject performing each activity 20 times with variations in execution.	N.A.	Door sensors, motion detectors, smart outlets, water-flow sensors.	Cooking, preparing a hot beverage, taking care of personal hygiene.
Arrotta et al. [71]	CNNs and three different XAI approaches: Grad-CAM, LIME, and Model Prototypes.	Self collected, CASAS.	A = 0.8 (min.), A = 0.9 (max).	Magnetic sensors on doors and drawers, pressure mat on chairs, smart-plug sensors for home appliances, smartwatches collecting inertial sensor data.	Phone answering, table clearing, hot meal cooking, eating, home entering, home leaving, phone calling, cold meal cooking, table setting, medicine taking, working, dish washing, TV watching.
Hanif et al. [73]	Neural network and KNN classifiers.	Self collected.	A = 0.99340162 (avg.).	Wrist wearable device and pocket positioned smart phone (accelerometer, gyroscope, magnetometer).	25 basic and complex human activities (e.g., eating, smoking, drinking, talking, etc.).
Syed et al. [75]	L4 Haar wavelet for feature extraction, 4-2-1 1D-SPP for summarization, and hierarchical KNN for classification.	SisFall.	F1S = 0.9467 (avg.).	2 accelerometers and 1 gyroscope placed on waist.	Slow/quick walking, slow/quick jogging, slow/quick stair-climbing, slow/quick chair sitting, chair collapsing, slow/quick lying-sitting, position changing (back-lateral-back), slow knee bending/straight standing, seated car entering/exiting, walking stumbling, high-object jumping.
Roberge et al. [74]	Supervised learning and MinMaxScaler, to recognize hand gestures from inertial data.	Self collected involving 21 subjects, made publicly available.	A = 0.83 (avg.).	Wristband equipped with a triaxial accelerometer and gyroscope.	Simple gestures for cooking activities, such as stirring, cutting, seasoning.
Syed et al. [82]	XGBoost algorithm.	LARa.	A = 0.7861 (avg.).	Accelerometer and gyroscope sensor readings on both the legs, arms and the chest/mid-body.	Standing, walking, cart walking, upwards handling (hand raised to shoulder), centered handling (no bending/lifting/kneeling), downwards handling (hands below knees, kneeling).
Muaaz et al. [81]	MDS, magnitude data, time- and frequency-domain features, feature-level fusion, SVM.	Self collected.	A = 0.915 (min.), A = 1.000 (max.).	IMU sensor placed on the lower back, Wi-fi receiver.	Walking, falling, sitting, picking up an object from the floor.
Wang et al. [78]	Clustering-based activity confusion index, data-driven approach, hierarchical activity recognition, confusion relationships.	UCI-HAR.	A = 0.8629 (min.), A = 0.9651 (max.).	Smartphone equipped with accelerometers and gyroscopes.	Standing, sitting, laying down, walking, downstairs/upstairs.
Fan et al. [79]	BSO, deep Q-network, hybrid feature selection methodology.	UCI-HAR, WISDM, UT_complex.	A = 0.9841 (avg.).	Inertial sensors integrated into mobile phones: one in the right pocket and the other on the right wrist to emulate one smartwatch.	Walking, jogging, sitting, standing, biking, using stairs, typing, drinking coffee, eating, giving a talk, smoking, with each carrying.
Sengul et al. [80]	Matrix time series, feature fusion, modified Better-than-the-Best Fusion (BB-Fus), stochastic gradient descent, optimal Decision Tree (DT) classifier, statistical pattern recognition, k- Nearest Neighbor (kNN), Support Vector Machine (SVM).	Self collected involving 20 subjects.	A = 0.9742 (min.), A = 0.9832 (max.).	Accelerometer and gyroscope integrated into smartwatches (Sony SWR50).	Being in a meeting, walking, driving with a motorized vehicle.
Chen et al. [83]	Received Signal Strength Indicator (RSSI), coarse-to-fine hierarchical classification, butterworth low-pass filter, and a SVM, GRU, RNN.	Self collected involving 6 subjects.	A = 0.9645 (avg.).	WiFi-RSSI sensors nodes.	Standing, sitting, sleeping, lying, walking, running.
Gu et al. [84]	CSI features, DL, dual-channel convolution-enhanced transformer.	Self collected involving 21 subjects.	A = 0.933 (min.), A = 0.969 (max.).	WiFi-CSI sensors nodes.	Walking, standing up, sitting down, fall to the left/right/front.
Wu et al. [85]	3D point cloud data, 3D radar, voxelization with a bounding box, CNN-Bi-LSTM classifier.	Self collected.	A = 0.9673 (avg.).	3D mmWave radars.	Food intake activities.
Qiao et al. [86]	Time-range-Doppler radar point clouds, convolutional multilinear principal component analysis.	Self collected involving 10 volunteers.	A = 0.9644 (avg.), P = 0.94 (avg.), R = 0.947 (avg.), SP = 0.988 (avg.), F1S = 0.944 (avg.).	Millimeter-wave frequency-modulated continuous waveform (FMCW) radar.	Normal walking, walking with plastic poles in both hands, jumping, falling down, sitting down, standing up.
Zhang et al. [87]	Energy domain ratio method, local tangent space alignment, adaptive extreme learning machine, multiangle entropy, improved extreme learning machine.	Self collected with one person tested at a time.	A = 0.86 (min.), A = 0.98 (max.).	Millimeter-wave radar operating at 77–79 GHz.	Chest expanding, standing walk (swing arm), standing long jump, left-right turn run, one-arm swing, alternating arms swing, alternating legs swing.
Li et al. [88]	Bidirectional LSTM (BiLSTM), Residual Dense-BiLSTM, Analog-to-digital converter, Adam optimizer, GPU processing.	Self collected involving 8 subjects.	A = 0.98 (avg.).	TENG-based gait sensor.	Standing, jogging, walking, running, jumping.
Zhong et al. [77]	Health parameters, Physical Activity Recognition and Monitoring, Support Vector Machine (SVM), Internet of Things.	Self collected involving 10 healthy students.	A = 0.98 (avg.).	Wearable ECG.	Sitting, standing, walking, jogging, jumping, biking, climbing, lying.

**Table 3:** Multimodality.

Reference	Methods	Dataset/s	Performance	Sensor/s	Actions
Xiao et al. [91]	Self-attention mechanism and the two-stream structure to model the temporal-spatial dependencies in multimodal environments.	PAMAP2, Opportunity, USC–HAD, and Skoda	F1S = 0.98 (PAMAP2), F1S = 0.69 (Opportunity), F1S = 0.57 (USC–HAD), F1S = 0.95 (Skoda).	IMU, accelerometer, gyroscope, magnetometer, and orientation sensor.	Walking, running, jumping, cycling, standing, sitting.
Bocus et al. [24]	Data collection from multiple sensors, in two furnished rooms with up to six subjects performing day-to-day activities. Validated using CNN.	Self collected, new publicly available dataset.	A = 0.935 (WiFi CSI), A = 0.865 (PWR), A = 0.858 (Kinect), A = 0.967 (data fusion).	WiFi CSI, Passive WiFi Radar, Kinect camera.	Sitting down on a chair, standing from the chair, laying down on the floor, standing from the floor, upper body rotation, walking.
Islam et al. [92]	CNNs, Convolutional LSTM.	UP-Fall	A = 0.9761	Accelerometers, gyroscopes, RGB cameras, ambient luminosity sensors, electroencephalograph headsets, and context-aware infrared sensors.	Walking, standing, sitting, picking up an object, jumping, laying, falling forward using hands, falling forward using knees, falling backwards, falling sideward, falling sitting in an empty chair.
Alexiadis et al. [93]	Feed-forward artificial neural network as the fusion model.	ExtraSensory	F1S = 0.86	Accelerometers, gyroscopes, magnetometers, watch compasses, and audio.	Sitting (toilet, eating, TV watching, cooking, cleaning), standing (cooking, eating, cleaning, TV watching, toilet), lying (TV watching, eating), walking (eating, TV watching, cooking, cleaning).
Dhekane et al. [94]	Similarity-based CPD, Sensor Distance Error (SDE), Feature Extraction, Classification, Noise Handling, Annotations.	CASAS (Aruba, Kyoto, Tulum, and Milan).	A = 0.9534 (min.), A = 0.9846 (max.).	Motion, light, door and temperature, associated with objects.	All included in Aruba, Kyoto, Tulum, and Milan.
Hiremath et al. [95]	Passive observation of raw sensor data, Representation Learning, Motif Learning and Discovery, Active Learning.	CASAS (Aruba, Milan)	A = 0.286 (min.), A = 0.944 (max.)	Door, motion, temperature.	Bathroom, bed to toilet, meal preparation, wash dishes, kitchen activity, eating, dining room activity, enter/leave home, relax, read, watch TV, sleep, meditate, work, desk activity, chores, house keeping, bedroom activity.
Abdel-Basset et al. [89]	Supervised dual-channel model, LSTM, temporal-spatial fusion, convolutional residual network.	UCI-HAR, WISDM.	A = 0.977 (avg.), F1S = 0.975 (avg.).	Accelerometer and gyroscope in wearable devices.	Upstairs, downstairs, walking, standing, sitting, laying, and jogging.
Gorjani et al. [90]	Multilayer perceptron, context awareness, single-user, long-term context, multimodal, data fusion.	Self collected involving 1 person for 7 days.	A = 0.912 (min.), A = 1.000 (max).	2 IMU (gyroscope, accelerometer, magnetometer, barometer) on the right hand and the right leg, KNX sensors for room temperature, humidity level (%), CO2 Concentration level (ppm).	Relaxing with minimal movements, using the computer, preparing tea/sandwich, eating breakfast, wiping the tables/vacuum cleaning, exercising using stationary bicycle.

**Table 4:** Real-time processing.

Reference	Methods	Dataset/s	Performance	Sensor/s	Actions
Zin et al. [10]	UV-disparity maps, spatial-temporal features, distance-based features, automatic rounding data fusion.	Self collected	A = 0.865 (min.), A = 1.000 (max.).	Depth cameras	Outside the room, transition, seated in the wheelchair, standing, sitting on the bed, lying on the bed, receiving assistance, falling.
Hu et al. [103]	Genetic algorithm-optimized SVM for real-time recognition.	CASAS	mF1S = 0.9	Motion and door sensors.	Bathe, bed–toilet transition, cook, eat, leaving, personal hygiene, sleep, toilet, wash dishes, work at table, and other activity.
Chen et al. [104]	OpenPose library for skeleton extraction, CNN classifier, real-time processing.	Self collected from real public places.	A = 0.973	RGB camera	Squat, stand, walk, and work.
Yan et al. [105]	Unsupervised feature learning, enhanced topic-aware Bayesian, HMM.	CASAS (Aruba, Cairo, Tulum, Hhl02, Hhl04).	A = 0.8357 (min.), F1S = 0.5667 (min.), A = 0.9900 (max.), F1S = 0.9807 (max.).	Environmental sensors (see CASAS).	Meal prepare, relax, eat, work, sleep, wash dishes, bed to toilet, enter home, leave home, housekeeping, resperate.
Ramos et al. [106]	Bidirectional LSTM networks, sliding window.	CASAS (Milan).	P = 0.90 (min.), P = 0.98 (max.), R = 0.88 (min.), R = 0.99 (max.), F1S = 0.89 (min.), F1S = 0.98 (max.).	Environmental sensors (see CASAS).	Bed to toilet, chores, desk activity, dining rm activity, eve meds, guest bathroom, kitchen activity, leave home, master bathroom, meditate, watch tv, sleep, read, morning meds, master bedroom activity, others.
Minarno et al. [96]	Logistic regression, multiuser, long-term context, lightweight model, and real-time processing.	UCI-HAR.	A = 0.98 (max.).	Triaxial accelerometer and gyroscope embedded in wearable devices.	Laying, Standing, Sitting, Walking, Walking Upstairs, Walking, Downstairs.
Maswadi et al. [97]	Naive Bayes (NB), DT, multiuser, long-term context, real-time processing.	UTD-MHAD.	A = 0.886 (min.), A = 0.999 (max.).	Accelerometer placed at four different locations: right arm, right biceps, waist, and belly.	Sitting, standing, walking, sitting down and standing up.
Thakur et al. [98]	Online CPD strategy, correlation-based feature selection, ensemble classifier.	UCI-SBHARPT.	A = 0.9983 (avg.).	Accelerometer and gyroscope sensors of a smartphone.	Walking, walking upstairs, walking downstairs), three static activities (sitting, standing, lying), and six transitional activities (stand-to-sit, sit-to-stand, sit-to-lie, lie-to-sit, stand-to-lie, lie-to-stand.

**Table 5:** Interoperability.

Reference	Methods	Dataset/s	Performance	Sensor/s	Actions
Zhang et al. [108]	Four-layer architecture (physical layer, middleware layer, knowledge management layer, service layer), Unordered Actions and Temporal Property of Activity (UTGIA), Agent Knowledge Learning.	Self collected in a lab setting simulating a smart home, involving 8 participants.	A = 0. 9922	Pressure, proximity, motion (ambient).	Take medicine, set kitchen table, make tea, make instant coffe, make hot drink, heat food, make sweet coffe, make cold drink.
Noor et al. [110]	Ontology, Protégé, reasoner.	Self collected in a lab setting, involving 20 participants; OPPORTUNITY.	A = 0.915	Burner sensor, water tap sensor, item sensor, chair sensor, drawer sensor, flush sensor, pir sensor, wearable sensors (accelerometers, gyroscopes, magnetometers).	Getting up, grooming, relaxing, coffee/sandwich prep and consumption, cleaning; door/drawer/fridge/dishwasher operation, light control, drinking in various positions.
Franco et al. [109]	ILM, IoT, HAN, FFNN, LSTM, SVM.	UK-DALE	A = 0.95385 (FFNN), A = 0.93846 (LSTM), A = 0.83077 (SVM).	Smart plugs.	Washing the dishes, baking food, ironing, drying hair, doing laundry, sleeping, unoccupied.
Stavropoulos et al. [72]	OWL, Sparql, Gaussian Mixture Model Clustering, Fisher Encoding, Support Vector Machine, Sequential Statistical Boundary Detection.	Self collected in a clinical setting, 98 participant trials.	R = 0.825, P = 0.808.	Ip camera, microphone, worn accelerometer, smart plugs, object-tag with accelerometer.	Answering phone, establishing account balance, preparing drink, preparing drug box.
Mekruksavanich et al. [111]	sEMG, MLP, DT.	Self collected involving 10 subjects.	P = 0.9852 (avg.), R = 0.9650 (avg.), A = 0.9900 (avg.).	Wearable sEMG.	Exercise activities.
Minarno et al. [112]	LG, SVM, KNN.	UCI-HAR.	A = 0.9846 (min.), A = 0.9896 (max.).	Accelerometer and a gyroscope embedded in a smartphone.	Sitting, standing, lying, walking, walking up/down stairs.
Javed et al. [107]	MLP, Integration with other smart-home systems, high-performance computing.	WISDM, Self collected.	A = 0.93 (avg.).	2-axis accelerometer worn in front pant’s leg pocket.	Standing, sitting, downstairs, walking, upstairs, jogging.
Muaaz et al. [113]	CNN, Wi-Fi CSI, spectrograms, health information systems (HIS).	Self collected involving 9 subjects.	A = 0.9778 (avg.).	Wi-Fi NICs in the 5 GHz band.	Walking, falling, sitting on a chair, picking up an object from the floor.

**Table 6:** Resource-Constrained Processing.

Reference	Methods	Dataset/s	Performance	Sensor/s	Actions
Zhou et al. [116]	Back-Dispersion Communication, Wearable Internet of Things, DL, Bayesian Networks, EDL, Variable Autoencoder, Convolution Layers.	Self collected.	A = 0.80 (min.), A = 0.97 (max.).	Wearable Sensor Network.	Walking.
Chang et al. [117]	IoT, ESVM, SVM, CNN, Raspberry Pi, STM32 Discovery Board, Tensorflow Lite, Valgrind Massif profiler.	MNIST [118].	A = 0.822, P = 0.8275, R = 0.8189, F1S = 82.32.	N.A.	N.A.
Zhu et al. [119]	STFT, CNN, ARM platform	Self collected.	A = 0.9784.	Infineon Sense2GoL Doppler radar.	Running, walking, walking while holding a stick, crawling, boxing while moving forward, boxing while standing in place, sitting still.
Helmi et al. [120]	SVM, RFC, RCN, RNN, BiGRU, MPA, PSO, DE, GSA, SSA.	Opportunity, PAMAP2, UniMiB-SHAR.	A = 0.8299 (min.), A = 0.9406 (max.).	Tri-axial accelerometer, gyroscope, magnetometer.	Open/close door/fridge/drawer, clear table, toggle switch, sip from cup, ADLs, fall actions.
Angerbauer et al. [121]	SVM, CNN, RNN.	UCI-HAR.	F1S=0.8834 (min.), F1S = 0.9562 (max.).	Accelerometer and gyroscope from a pocket-worn smartphones.	Standing, sitting, laying, walking, walking upstairs, walking downstairs.
Ahmed et al. [122]	SFFS, multiclass SVM.	UCI-HAR.	A = 0.9681 (avg.).	Accelerometer and gyroscope from a pocket-worn smartphones.	standing, sitting, lying, walking, walking downstairs, walking upstairs, stand-to-sit, sit-to-stand, sit-to-lie, lie-to-sit, stand-to-lie, lie-to-stand.
Imran et al. [114]	Convolutions Neural Network, multiuser, long-term context, multimodal, lightweight processing.	UCI-HAR, WISDM.	A = 0.925 (avg.).	Accelerometer and gyroscope in wearable devices.	Standing, sitting, lying, walking, walking upstairs, walking downstairs, jogging.
Betancourt et al. [115]	Self-attention network, multiuser, long-term context, multimodal, lightweight processing.	UCI-HAR, Self collected (publicly released with this study).	A = 0.97 (avg.).	Accelerometer, gyroscope, and magnetometer embedded in wearable devices.	Standing, sitting, lying, walking, walking upstairs, walking downstairs.

## Data Availability

Not applicable.

## Abbreviations

The following abbreviations are used in this manuscript:

AAL	Ambient Assisted Living
ADL	Activity of Daily Living
BiGRU	Bi-directional Gated Recurrent Unit
BSO	Bee Swarm Optimization
CNN	Convolutional Neural Network
CPD	Change Point Detection
CPI	Change Point Index
CSI	Channel State Information
DE	Differential Evolution
DL	Deep Learning
DT	Decision Tree
EAR	Exercise Activity Recognition
EDL	Enhanced Deep Learning
EMI	Electromagnetic Interference
ERT	Extremely Randomized Trees
FFNN	Feed-Forward Neural Network
GRU	Gated Recurrent Unit
GSA	Gravitational Search Algorithm
GTB	Gradient Tree Boosting
HAN	Home Area Network
HAR	Human Action Recognition
HMM	Hidden Markov Model
IBCN	Improved Bayesian Convolution Network
ILM	Intrusive Load Monitoring
IMU	Inertial Measuring Unit
ICT	Information and Communication Technology
IoT	Internet of Things
KNN	K-Nearest Neighbors
LR	Logistic Regression
LSTM	Long Short-Term Memory
LSVM	Linear Support Vector Machine
MDS	Time-variant mean Doppler shift
ML	Machine Learning
MLP	Multilayer perceptron
MPA	Marine Predator Algorithm
NIC	Network Interface Card
PIR	Passive Infrared
PSO	Particle Swarm Optimization
RCN	Residual Convolutional Network
RF	Radio Frequency
RFC	Random Forest Classifier
RGB	Red-Green-Blue
RiSH	Robot-integrated Smart Home
RNN	Recurrent Neural Network
SDE	Sensor Distance Error
SDR	Software Defined Radio
sEMG	Surface Electromyography
SFFS	Sequential Floating Forward Search
SKT	Skin Temperature
SSA	Salp Swarm Algorithm
STFT	Short-Time Fourier Transform
SVM	Support Vector Machine
TCN	Temporal Convolutional Network
TENG	Triboelectric Nanogenerator
TTN	Two-stream Transformer Network
UWB	Ultra-Wideband
W-IoT	Wearable Internet of Things
